# A comprehensive software suite for protein family construction and functional site prediction

**DOI:** 10.1371/journal.pone.0171758

**Published:** 2017-02-09

**Authors:** David Renfrew Haft, Daniel H. Haft

**Affiliations:** 1 J. Craig Venter Institute, Rockville, Maryland, United States of America; 2 National Center for Biotechnology Information, National Library of Medicine, National Institutes of Health, Bethesda, Maryland, United States of America; Universite Paris-Sud, FRANCE

## Abstract

In functionally diverse protein families, conservation in short signature regions may outperform full-length sequence comparisons for identifying proteins that belong to a subgroup within which one specific aspect of their function is conserved. The SIMBAL workflow (Sites Inferred by Metabolic Background Assertion Labeling) is a data-mining procedure for finding such signature regions. It begins by using clues from genomic context, such as co-occurrence or conserved gene neighborhoods, to build a useful training set from a large number of uncharacterized but mutually homologous proteins. When training set construction is successful, the YES partition is enriched in proteins that share function with the user’s query sequence, while the NO partition is depleted. A selected query sequence is then mined for short signature regions whose closest matches overwhelmingly favor proteins from the YES partition. High-scoring signature regions typically contain key residues critical to functional specificity, so proteins with the highest sequence similarity across these regions tend to share the same function. The SIMBAL algorithm was described previously, but significant manual effort, expertise, and a supporting software infrastructure were required to prepare the requisite training sets. Here, we describe a new, distributable software suite that speeds up and simplifies the process for using SIMBAL, most notably by providing tools that automate training set construction. These tools have broad utility for comparative genomics, allowing for flexible collection of proteins or protein domains based on genomic context as well as homology, a capability that can greatly assist in protein family construction. Armed with this new software suite, SIMBAL can serve as a fast and powerful *in silico* alternative to direct experimentation for characterizing proteins and their functional interactions.

## Introduction

Data-mining methods can very efficiently generate hypotheses that certain protein families work together to carry out some biological process. Historically, analysis then often gets “stuck” waiting for experimental testing that may not be forthcoming. SIMBAL [1] allows for follow-up investigation *in silico* once correlations have been noted between pairs of protein families. By showing which features in a protein appear to matter most, SIMBAL can deepen our understanding of how molecular function links one family of proteins to another. Given a training set in which homologs from a functionally diverse protein superfamily have been labeled either YES or NO, according to features in the genomes from which they were taken, SIMBAL can detect short signature regions for which the best BLAST matches skew overwhelmingly toward the YES set. If a solved crystal structure exists for the query protein or one of its homologs, mapping the signature region identified by SIMBAL onto the crystal structure can shine a light on the underlying biology.

In any newly sequenced genome, the functions of many proteins are unknown. Characterized or not, most proteins belong to some *subsystem[2, 3]*. In a subsystem, several components work together to carry out a biological process, such as biosynthesis of a cofactor, or import and utilization of a carbon source. HMM or BLAST searches readily find related proteins in different genomes, including homologs related closely enough to share a specific function. If such a functionally conserved protein is found in numerous species, other components of the subsystem(s) to which it belongs may be found in those species as well. This type of co-occurrence makes it possible for data-mining techniques such as Phylogenetic Profiling [4, 5], gene neighborhood analysis, operon detection, “Rosetta stone” gene fusion analysis, text mining, or several methods together, as in the STRING database [6], to identify sets of proteins that constitute previously undescribed subsystems, and that may carry out an undocumented biological process [7]. Unfortunately, there is a mismatch in speed, effort, and cost between generating the hypothesis that two families of proteins are connected through their roles in a subsystem—taking just seconds using bioinformatics methods—vs. the obvious follow-up laboratory work that might take years to set up and then complete. For one hypothesis at a time, the SIMBAL workflow lets an investigator use contextual clues from thousands of genomes to build a training set that will support further inquiry, and then use SIMBAL itself to search selected proteins for those short regions where highly similar amino acid sequences best reflect highly consistent genomic contexts. This purely *in silico* approach often yields confirmatory evidence for a functional connection between two proteins, plus new insights into functions and mechanisms, and may provide an attractive alternative to direct experimental assay.

This paper presents numerous software components that transform SIMBAL from an algorithm whose setup and execution require specialized knowledge and significant expenditures of effort into a simplified pipeline. While the various components of the software suite were developed primarily to assist with the operation of SIMBAL, several, most especially the Training Set Builder (TSB), can be of use for other types of work with protein families. The components of the software suite can be grouped into three categories—preparation of SIMBAL input, SIMBAL itself, and processing of SIMBAL output. The first category, input preparation, includes the Training Set Builder, the Domain Extractor, and a strongly recommended non-redundification step. The second category involves an improved version of SIMBAL, as well as the framework for a significantly faster parallelized-version of SIMBAL that can support being run on a grid infrastructure. The third category, output processing, involves R scripts for display of the data as triangular heatmaps, a re-weighting algorithm assisting in the visualization of signature regions, and software for extrapolating scores down to single residues.

Previous articles of ours introducing or invoking SIMBAL illustrate its flexibility and its power to highlight sequences that confer functional specificity. For example, the methyltransferase PrmC modifies a glutamine side chain in a specific target protein, PrfA (release factor 2). In many species, PrmC has functionally divergent paralogs that act on different target proteins. Several regions of sequence are rather well conserved among all true PrmC sequences. But one such region contains the enzyme’s active site, while another is involved in cofactor binding, so neither region readily distinguishes PrmC from the related methyltransferases that act on targets other than PrfA. When SIMBAL uses the contextual clue that certain methyltranferases were encoded close the *prfA* gene, its highest score goes instead to a third region, conserved in true PrmC sequences but not in its paralogs [1]. A co-crystal of PrmC with its PrfA target [8] shows that SIMBAL highlights a region critical for specificity, part of the PrmC:PrfA interface and close to the glutamine in its target that receives the methyl modification.

We previously showed that all species in which the GlyGly-CTERM putative protein-sorting signal occurs also encode a member of a particular branch of the rhomboid family of intramembrane serine proteases [9]. We hypothesized that proteases from that branch, which we named *rhombosortase*, were the cellular components responsible for recognizing and cleaving GlyGly-CTERM sequences. As we introduce the distributable SIMBAL software suite, we reprise SIMBAL’s portion of that computational study that shows rhombosortase is almost certainly a cleavage enzyme for the GlyGly-CTERM sorting signal. What once took weeks of effort and specialized knowledge to investigate was redone in a matter of hours, with only minimal manual involvement.

The potential uses of SIMBAL are many, including classifying cofactor-binding sites or substrate-binding sites in enzymes, locating protein-protein interaction sites, and confirming functional linkages between proteases and their targets. We have used the metabolic trait of urea catabolism to identify regions of ABC transporter permease subunits that appear to contribute to functional specificity (regions that form the substrate exit pore on the cytosolic face of the membrane) [1]. Other suitable uses for SIMBAL may remain to be discovered. SIMBAL is intended not to exempt researchers from benchwork, but to let observations of conserved genomic context made for large numbers of genomes become a part of the researcher’s tool kit for characterizing proteins.

The SIMBAL software suite is freely available under the GNU General Public License from https://github.com/SIMBAL2/SIMBAL_SOFTWARE_SUITE

## The SIMBAL software suite

### Training Set Builder

#### Overview

The Training Set Builder (TSB) collects members of a protein family of a user’s choosing (the *target* family), from a set of genomes provided by the user, and partitions the family based on the presence or absence of any number of other protein families (*attributes*) in those same genomes. The TSB allows the relative positions of the target and attribute families to be used in the partitioning process. Most commonly, position is considered when the average number of target family paralogs per genome is large, diluting the YES partition, but any paralog encoded sufficiently close to the attribute gene is likely belong to the same subsystem.

#### Obtaining genomes

The TSB can work off of locally stored genomes, or can download prokaryotic genomes from RefSeq. Downloaded genomes can be stored to improve the speed of future runs. Most commonly, the TSB will either be run on all Complete RefSeq genomes (-complete), or on all RefSeq genomes marked “reference” or “representative” (-reference) [10]. Other methods for selecting genomes, such as selecting specific species, or providing a user-specified list of accessions are also available, and are described in the usage guide included with the software.

For all genomes, the TSB requires at least the protein translation in FASTA format. If the positions of genes are also to be considered, the TSB also requires a “.gff” file. The TSB assumes that any file it cannot find locally is available, in compressed form, from the RefSeq collection of complete and high quality draft prokaryotic genomes. It will, as needed, go to RefSeq’s ftp site to download, uncompress, rename, and optionally keep the.fasta and.gff files required. We strongly recommend keeping and working with locally stored copies of these files, as on a typical system downloads are responsible for more than half of total runtime. The most common mode of operation is the -complete command line option, which allows the user to request specifically those genomes that RefSeq has marked as complete in their summary statistics. At this writing, 4776 of the 61146 genomes listed at ftp.ncbi.nlm.nih.gov/genomes/refseq/bacteria/assembly_summary.txt were marked complete. Similarly, the command line option, -reference allows the user to request specifically those genomes that RefSeq has marked as either reference or representative in their summary statistics, numbering 4978 genomes.

#### Working with molecular markers to build training sets

The TSB ignores existing functional annotation on proteins, and relies instead on protein profile hidden Markov models (HMMs), such as those available from the Pfam[11] and TIGRFAM [12] collections, or those custom-built by the user using the HMMER3 [13] package. Any gene whose corresponding protein scores above a user-specified cutoff to a given HMM serves as a molecular marker that can be used to direct the construction of training sets. Users obtaining HMMs from libraries such as Pfam can find cutoff values provided in the header section of the HMM itself. The “trusted cutoff” (TC) is the minimal score observed for proteins that beat the “gathering threshold” (GA), and it identifies trusted members of the family as defined by the HMM. The “noise cutoff” (NC) is the top score observed among proteins that fall below the gathering threshold and are considered non-members. Sequences whose score falls between TC and NC represent a gray area where the HMM does not say clearly if a member of the protein family was found or not. The TSB can (and should) simply ignore genomes that meet neither all criteria of a YES genome, nor all criteria of a NO genome, but instead fall into the gray area.

All proteins that match the target HMM are considered eligible for inclusion in the training set. These target proteins are then sorted based on the hits to the supplied attribute HMMs, according to the logical criteria defined by the user. This can be as simple as partitioning a single protein family based on the presence or absence of a second protein family, but may be as complex as desired by the user. Although functional annotation is not used in the partitioning process, the sequence files that constitute the created training set will still contain the protein product names that were assigned by RefSeq. The fidelity of annotations shown is not guaranteed, of course, but inspecting the annotations found in the YES and NO partitions may be a convenient way to see if a configuration file used by the TSB, or the individual HMMs involved, are behaving sensibly or if they need to be adjusted.

#### A configuration file directs training set construction

**Fig 1:**
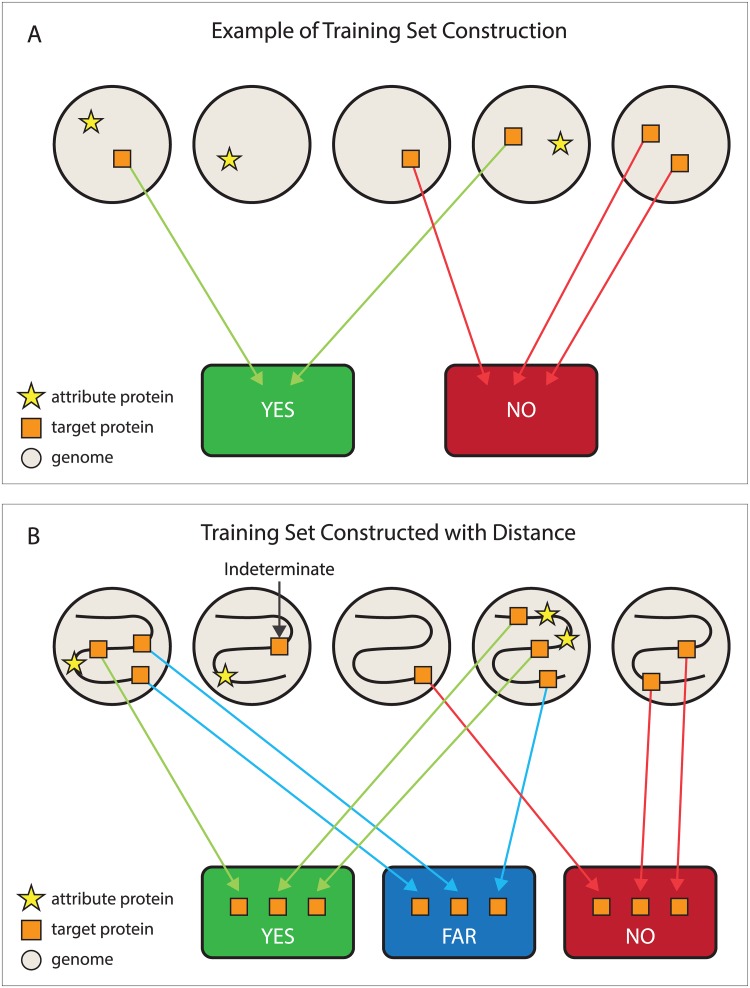
Training Set Construction. Fig 1A illustrates the simplest method for training set construction. Each genome (gray circles) is treated as a “bag of genes”; distance relationships between genes are ignored. One hidden Markov model (HMM) identifies target family proteins (orange squares) in the corresponding proteome. A second HMM finds proteins from a second family (yellow stars) whose presence or absence in the proteome is the attribute that controls how target family proteins are sorted. If an attribute family protein is found, members of the target family get sorted to the YES set (green container). If not, then target family proteins go to the NO set (red container). The training set builder (TSB) always works on one target protein family at a time, but more complicated rules may require multiple attributes to be jointly present for the YES set, and multiple attributes to be jointly absent for the NO set. Fig 1B shows training set construction using a distance rule. The S-shaped curved represents a long segment of genomic DNA. A target protein is sorted to the YES set if and only if its gene lies within a user-specified distance from the attribute protein’s gene. Target proteins from genomes that lack the attribute completely go to the NO set. A target protein goes to the FAR set if and only its gene sufficiently far from the nearest attribute gene, and the genome has already sent a target protein to the YES set. If a genome encodes an attribute family protein, but no target family protein qualifies for the YES set, then target family proteins are not sorted to any bin.

The user controls the partitioning performed by the TSB through a configuration file. In essence, the configuration file contains three pieces of information: the definition for the target family, the criteria for inclusion into the YES set, and the criteria for inclusion into the NO set. If a genome does not fully meet either set of conditions, target proteins from that genome are not included in the training set. Optionally, the criteria for the YES set can include a distance constraint. In these cases, if a genome meets the YES criteria, target family genes that meet the distance constraint are sorted into the YES set, while target family genes from the same genome that fail the distance constraint are sorted into the special FAR set. For a graphical depiction of the simplest case for training set construction both with and without distance constraints, see Fig 1.

#### An illustrative configuration file

Though described more fully in the file “CONFIG.README” provided alongside the software suite, here we present a sample configuration file. We’ve chosen as our example the configuration file used to update the analysis of the GlyGly-CTERM/rhombosortase system. The hypothesis that the enzyme we named rhombosortase [9] interacts with proteins that have a GlyGly-CTERM sequence region at their C-termini originated with our observation, made through a phylogenetic profiling method [5],that rhombosortases and GlyGly-CTERM-bearing sequences tended to be found in the same genomes. SIMBAL can be used to detect signature regions that best distinguish true rhombosortases from among all other rhomboid family intramembrane serine proteinases. In doing so, it shows that the essence of being a rhombosortase seems to lie in small number of residues deep in the substrate-binding cleft, an observation that makes sense to us only if this family of enzyme is specifically binding and cleaving GlyGly-CTERM proteins [9].

To perform this analysis, SIMBAL requires a YES set that is enriched in rhombosortases, and a NO set that is depleted in rhombosortases. The TSB produces these sets in an automated fashion by collecting all rhomboid family proteins (using HMM PF01694), and then partitioning them based on the presence or absence of GlyGly-CTERM protein sorting signals elsewhere in the contributing genome. We now know four HMMs that can identify proteins with a putative GlyGly-CTERM protein-sorting signal at the C-terminus: TIGR03501 (the general form of the GlyGly-CTERM signal), TIGR03867 (a variant form first seen in the *Ralstonia* lineage, and TIGR04179 and TIGR04212 (two full-length proteins that each end with a variant form of the GlyGly-CTERM sorting signal). These four “attributes” (GlyGly-CTERM sorting signals) can be used to sort the “targets” (rhomboid family proteins). The text shown below is the configuration file that represents this process and that was used to redo SIMBAL analysis of a rhombosortase sequence with the current set of available complete genomes.

TARGET: PFAM/PF01694.HMM

TARGET SCORE: 21.9

NO: TIGRFAM/TIGR03501.HMM<12.5

NO: TIGRFAM/TIGR03867.HMM<16

NO: TIGRFAM/TIGR04212.HMM<210

NO: TIGRFAM/TIGR04179.HMM<40

YES: (TIGRFAM/TIGR03501.HMM>16, TIGRFAM/TIGR03867.HMM>20, TIGRFAM/TIGR04212.HMM>295, TIGRFAM/TIGR04179.HMM>150)

This configuration file tells the TSB to collect all hits to PF01694. Rhomboid family proteases from genomes that hit any of the 4 markers (TIGR03501, TIGR03867, TIGR04212, TIGR04179) are sorted into the YES partition, while those from genomes that hit none of the 4 markers are sorted into the NO partition. Since the YES rules used the HMM trusted cutoffs, and the NO rules used the HMM noise cutoffs, it is possible that some genomes would be considered indeterminate. In such cases, the target family proteins from that genome are not included.

A search of 4776 complete genomes from RefSeq found in 204 genomes a GlyGly-CTERM protein scoring above the cutoffs specified in the configuration file. All of these genomes included at least one member of the rhomboid protease family. Thus, 547 proteins went to the YES partition of the rhomboid superfamily. The other *4572* genomes had no detectable GlyGly-CTERM protein. Of these, 3488 genomes contained 5364 members of PF01694, such as GlpG in *Escherichia coli*. Among YES genomes, there were on average 2.7 rhomboid family proteins per genome. Among NO genomes, there were on average 1.5 rhomboid family proteins per genome. Note that SIMBAL requires only a “training set”, not a “truth set.” There is no requirement that all members of the YES set have the desired function, and all members of the NO set lack it. In such a case, the problem would be solved in advance, and other techniques would be used to infer the importance of single residues. SIMBAL’s data mining algorithm can find very strong signals even if the majority of the YES set lacks the function in question, as long as the YES set is significantly enriched in proteins with the function relative to the NO set. Of course, once SIMBAL helps identify a short sequence strongly correlated with a particular function, the researcher is free to use searches based on that region to collect a smaller number of sequences that are all highly likely to function in the same way. For more details on the configuration files, program features, command line options, or usage instructions, please refer to the provided user guide.

#### Domain extraction tool

Proteins are collected by the TSB as full-length sequences, even if only one small region of the protein was recognized by the HMM and even if only that region is of interest to the analysis. If the collected proteins differ in their overall domain architecture, those differences could confound SIMBAL’s analysis. Therefore, we provide a domain extraction tool (DET). Studying domains individually may be advantageous in the case of large proteins in which individual domains have distinct functions, such as is the case with adenylation domains of non-ribosomal peptide synthases. The DET included in the software suite uses HMM search results to find the boundaries of each domain hit to the user-supplied HMM. Each domain (optionally with some additional flanking sequence) is exported as a distinct sequence, pulling multiple domains from a single protein if necessary, as in the case of proteins with repeats or duplications. The domain extracted needn’t be identified by the same HMM as was used to detect the target family. The TSB might use one HMM to collect a specific type of tRNA ligase, after which the DET could use a different HMM to extract their tRNA-binding domains.

When used as part of the SIMBAL workflow, domain extraction should precede non-redundification (see next section).

#### Non-redundification of training set sequences

When the TSB completes a run, the protein sets produced likely will contain extensive redundancy. This redundancy must be reduced; sequences that are identical or nearly identical should not be treated as independent observations. We recommend non-redundification such that the remaining sequences are never more than 80% identical.

We recommend use of the USEARCH utility from the UCLUST package[14] to identify one protein to keep for each cluster of highly similar sequences. USEARCH is available for free to academic/non-profit users, but its license prohibits redistribution. As such, we cannot directly make USEARCH available as part of the SIMBAL software suite, but as of writing, it is freely available for download from http://drive5.com/usearch/

An example of a non-redundification command, using USEARCH, is

usearch -cluster_fast YES_proteins.fasta -id 0.8 -centroids yes.clust.fasta

Alternatively, one may use CD-HIT [15] for the nonredundification step, or blastclust, part of the BLAST package and therefore already widely available, or some other package[16]. We provide a utility, *retain_by_blastclust*.*pl*, so users with blastclust properly installed may use a command such as

retain_nonredund.pl yes.fa 80 > yes80.fa

### SIMBAL

#### Running SIMBAL

In addition to the YES and NO (or YES and FAR) sets, the SIMBAL user must also supply a query sequence. Although the TSB builds a training set likely to contain a mix of true-positives and false-positives, and little confidence about protein function is needed to make an assignment to the YES partition, the choice of a query sequence is more critical. If the query protein selected does not have the function that the training sequence was built to study, then its sequence should not contain sites strongly correlated with the function in question, and SIMBAL should report no strong statistical signals, as compared to scores from sequences with the function. Getting a sense of the appearance of background results from SIMBAL runs is important for proper interpretation. The user should try a few different query sequences in order to show that SIMBAL locates its high-scoring regions consistently, and should run some expected negative examples, to show the contrasts between different query sequences as well as within a query sequence. Comparing SIMBAL results for a high-scoring query sequence vs. all its lower-scoring paralogs from the same genome, on the same color scale, can be especially instructive, and is strongly recommended.

The details of the SIMBAL algorithm were given in the original SIMBAL paper [1], but a simplified summary is included here for convenience. SIMBAL combines the sequences of the YES and NO partitions into one BLAST-searchable database, but labels each protein according to its source partition. SIMBAL scores each subsequence of the provided query sequence based on its ability to distinguish between the YES and NO sequences. To compute a score for a given subsequence, SIMBAL performs a BLAST search against the training set, then scans down the list of best hits in order. At each point in this scan, SIMBAL compares the relative numbers YES vs. NO sequences encountered so far, and computes the odds according to the binomial distribution that a random sampling of sequences from the training set could have yielded as many or more YES sequences. The score reported for a given subsequence is the negative log of the lowest probability (*i*.*e*. the greatest statistical signifance) computed at any point in the scan down the list of BLAST hits. Because more closely related sequences may be more likely to sort to the same partition, the occurrences of YES and NO labels in the BLAST results lack true independence from factors other than protein function. Therefore, the user is cautioned that SIMBAL scores should be used for comparing different regions of query sequence to each other, but not as a true measure of statistical significance.

The SIMBAL scoring algorithm is largely unchanged since its original description, but was updated to use NCBI BLAST+ instead of the proprietary WU-BLAST, and given improved handling for certain edge cases. Having removed dependencies on proprietary software, we now make SIMBAL itself available for distribution for the first time. Previously, it ran only on a (now defunct) server, and only at a coarse-grained resolution so as to limit the computational costs. Retesting examples presented in previous SIMBAL papers, using the new selection of representative genomes from RefSeq, the TSB to prepare training sets in automated fashion, and the revised SIMBAL software, gives equivalent results. For those interested in more specifics of the SIMBAL algorithm itself, the details can be found in the original SIMBAL publication [1].

#### Computational speed *vs*. resolution

**Fig 2:**
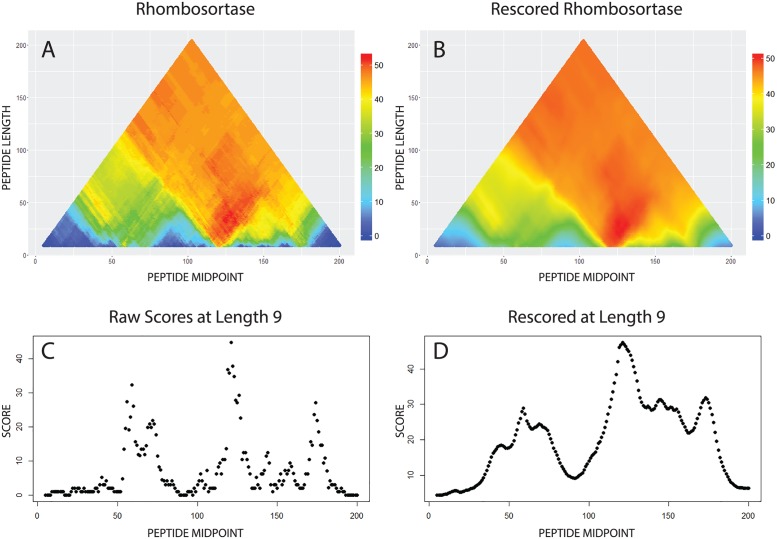
Graphical output from SIMBAL computation and post-processing. Fig 2A shows the triangle heat map obtained for query sequence NP_718091.1, a rhombosortase from *Shewanella oneidensis* MR-1, obtained as described in the text. Each colored pixel in the heat map conveys three pieces of information: a SIMBAL score (color, where red indicates greater statistical significance), the length of the subsequence being scores (height on the Y-axis), and the location of the middle of the subsequence along the length of the complete protein (position on the X-axis). Subsequences are evaluated from a minimum length of 9 (bottom of the heat map) to a maximum of 204, the full length of the protein, at the top of the heatmap. Scores are computed as the negative log_10_ of the odds against encountering, purely by chance, at least as great a preponderance of YES set-derived sequences among the top BLAST hits. Note that nearby pixels may differ sharply in score, and that the deepest red colors appear in a “plume” of pixels whose corresponding subsequences all contain the same key small region. Fig 2**B** shows a smoothed heatmap that results from re-processing SIMBAL scores so that each pixel represents a blend of its own score and those of longer sequences that contain it, performed iteratively starting with the longest sequences. The heritability parameter used was 0.93. Fig 2**C** shows data in numerical form corresponding to a line passing through the heatmap of Fig 2**A** very near its base, at a subsequence length of 9, with height rather than color showing the score. Fig 2**D** shows the corresponding slice through the rescored heatmap of Fig 2B, with a greatly reduced jitter in scores from one pixel to the next, and a clear indication of which short subsequences most likely contain key sites that discriminate rhombosortases from other rhomboid family proteases.

SIMBAL runtime is directly proportional to the total number of BLAST searches performed, and therefore depends on the length of the longest subsequence examined, the spacing between subsequences examined, and the number of different lengths of subsequences examined. The small size of the training set, compared to comprehensive protein sequence databases, makes each search fast and minimizes the noise of spurious matches to unrelated sequences. Once the user has selected a query sequence, instructing SIMBAL to inspect every possible subsequence length (-j 1) and every possible position (-w 1) will give the highest possible resolution. Using runtime parameters that instead sample only every third possible subsequence length (-j 3) and every third possible center position (-w 3) at a given length will produce a nine-fold increase in speed, at some cost in resolution. Our rhombosortase example used SO_2504 (NP_718091.1) as a query sequence. The results are represented graphically in Fig 2. The fine-grained analysis required 19,900 BLAST searches for subsequences of every length from 6 to the full sequence length of 204, at every position, against a training set containing 180 YES and 1879 NO sequences. The complete run took 4 hours on a 2-processor host.

The SIMBAL algorithm lends itself easily to parallelization, and the existing software is designed to accommodate this possibility. Since each institution tends to have a different grid setup, we cannot provide a pre-made parallelized version, but we do provide an explanation for how one might implement a parallelized version of SIMBAL, as well as a sample wrapper script. With parallelization, it is possible for a full-resolution run that would take 8+ hours using the standard version of SIMBAL to instead take only 10 minutes.

#### Post-processing SIMBAL results for visualization and interpretation

SIMBAL produces tab-delimited output that is designed to be easy to parse. The software suite provides tools written in ***R*** that perform post-processing of SIMBAL output in order to present the results graphically, and in order to help users interpret SIMBAL output and identify which few amino acids may play the greatest role in conferring functional specificity. ***R*** is freely available under the GNU general public license.

#### Visualizing SIMBAL results as triangular heat maps

The recommended graphical display of SIMBAL data is with what we call a “triangular heat map”. The triangular heat map is a tri-variate scatter plot, where the x-axis represents the midpoint of each scored subsequence, the y-axis represents the length of each scored subsequence, and the color of each data point represents the SIMBAL score for that subsequence. The shortest subsequences, represented at the bottom of the heat map, can have their midpoint almost anywhere along the query sequence. By contrast, the full-length sequence, at the top of the heat map, has only one possible placement. The result is that heat maps are triangular if all subsequences are searched, or trapezoidal if longer sequences are not explored. Point size is set to create the appearance of a continuous triangular object rather than a scatter plot. With such a graphical representation, hot spots corresponding to signature regions (if present), are readily detectable.

To improve the clarity of this graphical display, some adjustments were made. Because purple appears similar to both blue and red, we opted to display our data using a “short rainbow”; blue corresponds to the lowest score in the provided data set, while red corresponds to the highest score or to a user-specified maximum value. A function that converts scaled scores into RGB values ranging from (255,0,0) to (0,0,255) was written to accomplish this task. A second adjustment was performed to further improve the quality of the color display. As a wide range of wavelengths is interpreted by human color vision as uniformly green, a one-to-one mapping of scores to movement along a color spectrum, while mathematically accurate, actually provides for a misleading coloring. To compensate for this effect, instead of moving through the spectrum at a uniform rate, we move through different portions of the spectrum at different rates, to create a coloring scheme where uniform changes of value more closely correspond to uniform changes in color appearance. We judged the colors produced by this function to be more visually appealing, and easier to interpret, than any of the color palettes provided by default in ***R***. The triangular heat map for our rhombosortase example, using the improved color rendering, is shown in Fig 2.

#### Interpreting “plumes” in SIMBAL heat maps

For our discussion of the interpretation of SIMBAL heat maps, we introduce the term “plume”, which is an emergent property seen in many triangular heat maps. When a small group of nearby residues contributes significantly to the scores of sequences containing the group, they boost scores for a V-shaped region of the heat map, where the base of the V points to their location along the query sequence. That region will appear as a red plume. But even if all long subsequences containing the critical region are high-scoring, and show a bold red in the heat map, the base of the plume may score much more poorly, simply because BLAST searches conducted with very short sequences may not give fully meaningful results [17]. Consequently, the highest scoring sequences anywhere in the SIMBAL heat map tend to be too long to call our attention to precisely those few key residues likely to have the greatest role in dictating functional specificity. While the longer, top-scoring sequences high up in the plume better distinguish YES set proteins from NO set proteins by BLAST searches, and may serve as useful classifiers grouping proteins by function, users would benefit from a means to blend the strong signals found higher up in a plume with the more precise localization given by the shorter sequences at the base of the plume. Here, we provide two additional post-processing tools that use information from plumes rising above short sequences to assist in spotting the key residues that underlie high SIMBAL scores.

#### Re-scoring SIMBAL output

The first tool for further post-processing is an inheritance function that replaces the score at each point with a weighted average of a subsequence’s own raw score, and those of its two minimally longer “parent” sequences, where those sequences in turn have scores based in part on the scores of their parent sequences. The inheritance scheme results in a smoothing of the color map, but more importantly, it lets a short sequence gain a higher score if all sequences that contain it also score well. The reprocessing is run iteratively starting from the longest sequences in the heat map down to the shortest. This scheme serves to combat somewhat the penalty suffered by the shortest sequences, while also reducing overall noise. This results in a clearer image that somewhat highlights the base of plumes. The rescored heat map for our rhombosortase example is shown in Fig 2B. Panels **C** and **D** in Fig 2 show the scores at length 9 for rhombosortase before and after rescoring. Both traces correctly find the functional site inferred from prior crystallographic studies of related proteins, close to the active site Ser and presumably involved in substrate binding [9].

The second tool for further post-processing of SIMBAL output is a script that extrapolates scores down to a single residue by averaging all parent sequences of a user-specified fixed length. For instance, a given residue will be a subsequence in up to 30 sequences of length 30. Averaging across all of those gives a way to approximate the score for a given residue. The residues identified by this algorithm can help a user make sense of SIMBAL results, but users should note that this extrapolation down to single residues is simply an approximation, and not a score reported by SIMBAL itself. SIMBAL makes no assertion about sufficiently short sequences, since SIMBAL uses BLAST at the heart of its scoring mechanism, and for very short sequences, BLAST results become meaningless. The comparisons of values computed for single residues can be helpful, but should be treated with caution. For further examination of the roles of individual residues, it would be better to examine multiple sequence alignments, molecular phylogenetic trees, and/or crystal structures (if available), using SIMBAL to identify which portions of the sequence merit close attention. SIMBAL is intended to supplement rather than replace other comparative genomics tools.

Table 1 shows the sequential steps one may follow with the SIMBAL software suite, once the researcher has formed the hypothesis that genomic context helps predict which proteins in the target family share a key aspect of their function with a well-chosen query protein.

**Table 1:** Sequential steps in a SIMBAL analysis.

	Task	Tool	Inputs	Outputs
1	For the chosen target protein family, build the partitions that SIMBAL will need for its data-mining	**Training Set Builder**	Configuration file Genome list (defaults to RefSeq list)	**YES** fasta file **NO** fasta file **FAR** fasta file (if distance constraints are specified)
2	Restrict analysis to targeted domain (*optional*)	**Domain Extractor**	any fasta file any HMM	fasta file of the specified homology regions
3	Make each partition of the training set non-redundant (*optional*, *but strongly recommended*)	3^rd^ party tool, *e*.*g*. blastclust (wrapper script provided) or USEARCH	any fasta file	fasta file from which redundant sequences have been removed
4	Scan a selected query protein for subsequences whose best matches by BLAST skew overwhelmingly to the **YES** partition	**SIMBAL**	YES fasta file NO fasta file query sequence	SIMBAL output file
5	Reprocess SIMBAL output to assist in the localization of key sites (*optional*)	**Plume Finder**	SIMBAL output file	Rescored SIMBAL output file
6	Visually display SIMBAL results as a triangular heat map	**Heatmap Builder**	SIMBAL output file	Graphical display—triangular heatmap

#### Using the TSB to construct new protein families

Preparing SIMBAL runs is not the only use for the TSB. We have described the YES set as being enriched in proteins thought to match the query sequence in function, but not as having that function exclusively. In the rhombosortase example, fewer than 40% of the rhomboid family proteases in the YES set were examples of rhombosortase itself. Once the YES partition is built, several steps remain before the true rhombosortases can be identified and then used as the basis for building a protein family. Describing methods for building new protein families is outside the scope of this paper, but for some subsystems, the underlying biology lets the TSB be highly effective in choosing a good set of representative sequences for the defining multiple sequence alignment.

We tested the ability of the TSB to assist in protein family construction by reprising a study of the SCIFF biosynthesis subsystem [18]. SCIFF (“six cysteines in forty-five residues”) is a ribosomally translated, post-translationally modified peptide natural product, or RiPP [19, 20]. Its small size makes it easy to miss during structural annotation, although once found, it is easily recognized by TIGRFAMs model TIGR03973. The enzyme that modifies SCIFF is the radical SAM enzyme SCIFF maturase, or ScfB, a protein that can be identified by TIGRFAMs model TIGR03974. It is common for a single genome to contain 30 or more different radical SAM enzymes, all detectable by Pfam’s hidden Markov model PF04055 [11] but most of them uncharacterized, so classifiers that resolve them into functionally uniform subfamilies are needed to enable better genome annotation. We created a very simple distance-based configuration file:

TARGET: PFAM/PF04055.HMM

TARGET SCORE: 29.4

YES: ([500] TIGRFAM/TIGR03973.HMM>35)

NO: TIGRFAM/TIGR03973.HMM<35

The training set builder identified 234 examples of a radical SAM protein-encoding gene located within 500 base pairs of SCIFF precursor peptide-encoding gene. All 234 scored well above the cutoffs of the SCIFF maturase model, TIGR03974. The sequences found by the TSB are both more numerous and more diverse than those used six years ago to build TIGR03974. Given a new subsystem as pure as the SCIFF system (in which every precursor ScfA should be accompanied by a maturase ScfB), the training set builder can leverage one highly trustworthy molecular marker (the ScfA family, in which all clear homologs appear to be functionally equivalent) to pull a subfamily of interest from a superfamily as complex as radical SAM and build a new and equally value protein family definition.

## Discussion

SIMBAL was conceived of as a tool that performs one part of a multi-step *bioinformatics journey*, in which some computational method such as Partial Phylogenetic Profiling (PPP) [5] or STRING [6] has suggested to the researcher that two or more families of proteins cooperate in some biological process. The need for SIMBAL arises because the simple guilt-by-association hypothesis that there is a meaningful functional interaction between two families of proteins could be countered by any number of alternative hypotheses. The frequent co-occurrence of the F_420_ biosynthesis pathway with expanded numbers of luciferase-like monooxygenase (LLM) homologs suggests, for example, that F_420_ and not FMN is the cofactor these enzymes require and use [21]. But it might be instead that a single environmental feature coincidentally favors both F_420_ biosynthesis and the LLM family, or that certain members of the LLM family utilize metabolites that an unrelated F_420_-dependent enzyme helps make available. However, all these alternative explanations became far less tenable, and our primary hypothesis of abundant F_420_-dependent enzymes in *Mycobacterium* became very well supported, once SIMBAL analysis revealed that the plumes with the highest scores, from multiple paralogs, all centered on sites critical to cofactor-binding specificity [21]. Researchers may still demand direct experimental evidence for computationally derived conclusions before considering the story complete, but the power of SIMBAL’s data mining can bring an investigation along far enough to warrant the publication of some fairly richly developed and well-supported hypotheses.

The target family for SIMBAL analysis may have very few characterized proteins in it, or perhaps none at all. SIMBAL relies instead on the fact that while the co-occurrence of two protein families in any one genome is a very weak piece of evidence of a direct functional interaction, the combined weight of similar observations over tens, hundreds, or thousands of genomes may be very strong evidence indeed. SIMBAL’s examination of sites within proteins is qualitatively different from the post-homology methods that first group proteins into bins and that by design ignore the fine details of how sequences within a bin may be similar or different. Using an approach different from and largely orthogonal to the post-homology methods, by looking at sites within proteins, SIMBAL gets access to qualitatively different information. Its findings may add power or fine detail to preliminary findings that come from comparative genomics analyses and therefore may serve as a purely computational method that substitutes, in part, for direct experimental studies [22].

Previously, it was difficult to build a workflow that incorporated SIMBAL, as it required significant manual intervention to produce the necessary input, and required access to a now-defunct webserver. The software suite we present in this paper is fully distributable. It requires no access to relational databases or pre-computed data. It automates the preparation of SIMBAL training sets so that SIMBAL can now be used quickly and easily. Now that the preparation of training sets is automated, SIMBAL can reasonably be used in an exploratory manner, testing new hypotheses, rather than only following up in cases where a particular result is already strongly anticipated. Hypothesis generation via bioinformatics methods has always been relatively cheap. For the first time, using SIMBAL to follow up on these hypotheses is also cheap, allowing for exploration using SIMBAL—something not previously viable.

The tools designed for constructing SIMBAL training sets have broad utility, of which preparation of SIMBAL training sets is just one. While called a Training Set Builder, due to its usage in the SIMBAL workflow, the TSB is in fact a versatile tool for rule-based sequence collection. For those interested in constructing new Hidden Markov Models, the TSB represents a fast and easy way to collect large numbers of sequences that meet specific criteria, either for deciding on cutoff scores for newly created models, or for collecting sequences to be used as the seed for the model. The TSB preserves existing functional annotation as it collects sequences, allowing users to see how a model or theory stacks up against existing knowledge. For those interested in quality control in protein family construction, in genome assembly and gene-finding, or understanding of a pathway, the TSB makes it very easy to compare the behaviors of two or more linked protein families. Configuration files can be constructed for situations expected not to occur in Nature, and very quickly either show that available genome sequences confirm expectations or identify the set of genomes that are exceptions. Creative users will doubtless invent use cases for the TSB that were not anticipated when the software was being written. Bioinformatics researchers can now access incredible depths of genomic sequence data. This SIMBAL software suite provides powerful *in silico* tools so that we can better make sense of the data we’ve already collected.
